# Analysis of the effects of manipulating the space and number of players in small-sided games on the external load demands of futsal athletes in different age categories

**DOI:** 10.5114/biolsport.2025.145917

**Published:** 2025-01-14

**Authors:** Leandro Lume Gomes, Sérgio Adriano Gomes, Bruno Travassos, João Nuno Ribeiro, Iván Asín-Izquierdo, Henrique de Oliveira Castro, Carlos Ernesto Santos Ferreira

**Affiliations:** 1 Physical Education Faculty, Universidade de Brasília, Brasília, Brazil; 2 Department of Physical Education, Catholic University of Brasília, Brasília, Brazil; 3 Department of Physical Education, University Center of Brasília, Brasília, Brazil; 4 Secretaria de Estado de Educação do Distrito Federal, Brasília, Brazil; 5 Sport Science Department, Universidade da Beira Interior, Covilhã, Portugal; 6 Centro de Investigação em Desporto, Saúde e Desenvolvimento Humano – CIDESD, Covilhã, Portugal; 7 Portugal Football School, Federação Portuguesa de Futebol, Oeiras, Portugal; 8 Polytechnic Institute of Guarda, School of Education, Communication and Sports, Guarda, Portugal; 9 Sport Physical Activity and Health Research & Innovation Center – SPRINT, Portugal; 10 ENFYRED Research Group, Department of Musical, Plastic and Corporal Expression, Faculty of Social and Human Sciences, University of Zaragoza, Teruel, Spain; 11 Science-Based Training Research Group, Physical Performance and Sports Research Center, Department of Sports and Computer Sciences, Faculty of Sport Sciences, Pablo de Olavide University, Seville, Spain; 12 Football Science Institute, FSI Lab, Granada, Spain; 13 Grupo de Estudos e Pesquisas em Educação Física e Esportes, Physical Education Department, Universidade Federal de Mato Grosso, Cuiabá, Brazil

**Keywords:** Team sports, Ecological approaches, Nonlinear pedagogy, Constraints-led approach, Game manipulations, Conditioning games

## Abstract

The study aim was to analyse the effects of manipulating the space and number of players in small-sided games on the external load demands of futsal athletes of different age categories. Fifty-six male futsal players from U15, U17, U19, and adult age categories participated. The study lasted 7 weeks. The first week was for familiarization and evaluations to characterize the sample. In the six subsequent weeks there were six futsal tasks T1 (GK + 2 × 2 + GK, 20 × 20 m), T2 (GK + 2 × 2 + GK, 40 × 20 m), T3 (GK + 3 × 3 + GK, 20 × 20 m), T4 (GK + 3 × 3 + GK, 40 × 20 m), T5 (GK + 4 × 4 + GK, 20 × 20 m), and T6 (GK + 4 × 4 + GK, 40 × 20 m). The external load variables were measured with an inertial measurement unit (IMU), and kinematic (total and relative distance) and mechanical (total distance at high intensity accelerations and decelerations) data were extracted. T2 (133 m^2^) showed higher values for total distance covered (1248.0 ± 97.6 m) and distances covered at speeds between 12.1 and 18 km/h (407.8 ± 44.7 m) and greater than 18.1 km/h (115.2 ± 28.9 m) compared to T5 (40 m^2^) and T6 (80 m^2^). In addition, we observed greater variability in high-intensity accelerations and high-intensity decelerations, suggesting that regardless of field size and number of players, relative area might not play as crucial a role in this dimension.

## INTRODUCTION

In team sports such as futsal, the process of monitoring has allowed the analysis of player performance needs according to the game demands [1–3]. This process facilitates the adjustment and prescription of training exercises in an optimal and individualized way in relation to the competition [4]. Previous studies in futsal have used various tools, including video analysis, local positioning systems (LPS), ultra-wideband (UWB) tracking system technology, and inertial data to further understand the game demands and training procedures [3–6]. The devices available today do not require complex measurement protocols or indoor receiving antennas, and they can provide valid and reliable data adjusted to the needs of futsal [7]. With these systems, it is possible to measure and understand the relationship between internal and external load in training and competition.

The study of training tasks, mainly focused on small-sided games (SSGs) as a common type of task in the training programmes of sports teams, such as futsal, has been developed at different performance levels and age or gender categories [5, 8–10]. These tasks allow manipulation of a multitude of constraints in their design by modifying the movement patterns and responses of athletes [5, 8–12]. During training sessions, the analysis of small-sided games (SSGs) contributed to better understanding of the manipulation of different task constraints on players’ acute and chronic preparation in different sports [8, 9]. These results allow coaches to design training tasks according to the objective set in relation to specific individual and collective behaviours and requirements, without distorting the competitive reality [2, 11–13]. These types of activities favour the acquisition of movement skills, interpersonal coordination, and decision-making processes during training in relation to competition [14].

Futsal is a sport with a particular game dynamic based on alternating high-intensity efforts. In this sense, the format of the training tasks causes requirements that must be adjusted according to the demands of the competition. Accordingly, the main constraints manipulated in SSGs in futsal and football are space, time, number of players, rules, and feedback [8, 9, 15]. The playing space stands out as a key variable to be controlled in the design of SSGs. Larger playing areas are associated with greater distances covered, higher speeds achieved and greater volume of high-intensity actions, while smaller playing areas lead to an increase in the frequency of accelerations and decelerations [16–17]. However, these findings have not been tested in futsal, although other studies have observed that futsal players show a greater response in external loading variables during SSGs than soccer players [18]. For this reason, we could understand that these results could be reproduced even with greater differences depending on the playing space. In addition, small areas tend to promote a greater number of tactical and technical actions [19, 20]. The number of participant players also influences the internal and external load demands, with a lower number of players associated with higher demands (distance covered and distance at high speed, accelerating and decelerating distance at different ranges, Player Load, rating of perceived exertion (RPE), and heart rate), and a greater number of interventions per player (passing, dribbling, and shooting at goal) [5, 10]. Furthermore, a smaller number of players (e.g., 1 vs. 1) encourages dribbling and shooting actions, while a greater number of players promotes passing and ball retention actions with collective tactical behaviour [10]. Therefore, appropriate interventions through SSGs can effectively enhance physical, psychological, and technical/tactical performance simultaneously [5, 11, 21].

Despite previous studies in futsal which analysed the effects of the manipulation of SSGs [10, 18] or competitive demands [3, 4, 22, 23], there is a noticeable scarcity of references regarding the comparison between the GK + 4 vs. 4 + GK setup in 40 × 20 m with other SSGs. This deficiency makes it difficult to understand futsal training tasks in relation to competition. The only precedent, reported by Gomes et al. [5], showed that reducing the number of players relative to the competition structure increased the internal-external load demands and technical-tactical actions. In addition, age-category differences in external load responses to various SSGs have not been thoroughly explored in previous futsal references. In other sports, such as football, task analyses on external load by age categories have been carried out. However, in indoor sports such as futsal and basketball, studies have focused on internal load and technical-tactical aspects [24, 25].

Thus, the current investigation aimed to assess how manipulating the space and number of players in SSGs affects the external load demands on futsal athletes across different age groups. To the best of our knowledge, this study marks the first attempt to investigate the impact of space constraints, player numbers, competition structure, and various age categories in futsal. It was expected that decreasing player numbers and expanding activity space may heighten players’ external load, with variations across different age categories. These variations could be based on some tendency for the external load response to increase, although it is possible that the senior category sets a special context for the performance of the group. The study of SSGs in futsal, especially in relation to external loading, is still very limited and needs to be further expanded for application by futsal coaches.

## MATERIALS AND METHODS

### Sample

The sample size was calculated using G*Power version 3.1.9.7 (Heinrich-Heine Universität Düsseldorf – Düsseldorf, Germany), using the paired t-test with the following input parameters: (i) one-tailed (based on pilot study data); (ii) large effect size (≥ 0.50) as described by Cohen [26]; (iii) α = 0.05; and (iv) β = 0.95, and according to Field [27], indicating a minimum sample size of 20 individuals. The participants were recruited by convenience, according to the agreement of the club and the coaches to participate in the study. The inclusion criteria were based on the availability of the club and the coach to participate in the study. Moreover, only players who were part of the team age level in analysis, who did not report any injury, and who participated in all data collection were included in the study. The exclusion criteria adopted were athletes who suffered an injury that prevented them from training or did not participate in at least one of the data collection moments. The sample included 56 male futsal players from four categories (U15, U17, U19, and Seniors) (mean age = 18.23 ± 5.72 years; mean height = 1.70 ± 0.07 m; mean body mass = 63.44 ± 11.71 kg; mean % body fat = 11.88 ± 6.12%) from a club competing in the Castelo Branco (Portugal) district championship. The participants could be considered as tier 2 and 3 [28]. The sample of fourteen players per category had the following characteristics: U15 (13.6 ± 0.5 years; 1.70 ± 0.01 m; 55.3 ± 12.3 kg; 10.9 ± 7.6 %BF); U17 (15.7 ± 0.5 years; 1.7 ± 0.1 m; 62.2 ± 11.8 kg; 10.4 ± 4.9 %BF); U19 (17.3 ± 0.8 years; 1.7 ± 0.1 m; 64.8 ± 9.0 kg; 12.9 ± 6.5 %BF); Seniors (26.4 ± 5.8 years; 1.7 ± 0.2 m; 71.6 ± 8.1 kg; 13.3 ± 5.4 %BF). In terms of tactical positions, 14.29% were goalkeepers (GKs; n = 8), 17.86% were defenders (n = 10), 51.78% were wingers (n = 29), and 16.07% were pivots (n = 9). In terms of lower limb dominance, most of the players were right side dominant (82.14%).

Informed and written consent was provided by the club, the head coach, the players, and their legal guardians before the start of the data collection. The study protocol adhered to the guidelines of the ethics committee of the local university and the recommendations of the Declaration of Helsinki. The study protocol was approved by the Ethics Committee of the University of Beira Interior (CE-UBIPj-2018-029).

### Experimental design

**FIG. 1:**
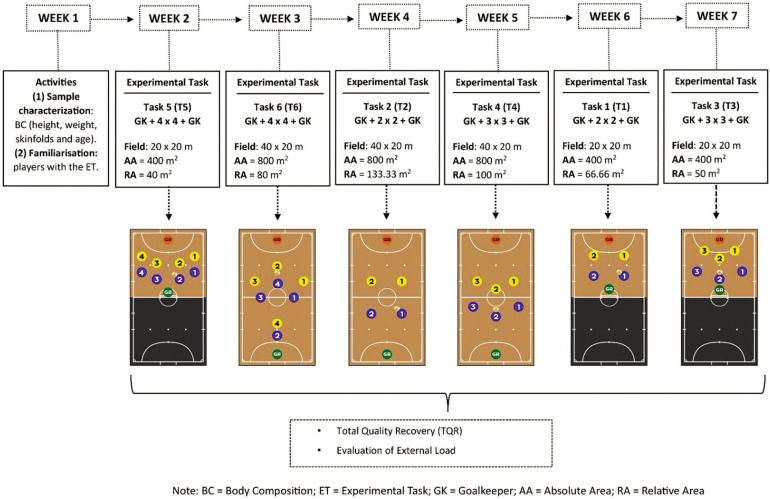
Study design

This cross-sectional study lasted 7 weeks and was carried out between October and December 2023, during the middle phase of the competition season (Figure 1). In the first week, participants were familiarized with the intervention procedures, and evaluations were carried out to characterize the players. In the following six weeks, the players in the four categories performed the six futsal tasks (T1, T2, T3, T4, T5, and T6). T6 was a futsal game played under the official rules of the sport [29], while different task constraints were manipulated in the other tasks (T1–T5) including: (i) the number of players involved in the task; (ii) dimensions of the playing field in terms of absolute area (AA = length × width) and relative area (RA = AA ÷ number of players involved in the task); (iii) all the tasks were carried out in the presence of GKs, who defended official goals with dimensions of 3 × 2 m (width and height, respectively); and (iv) tactical and technical actions: (a) attacking phase – the number of contacts with the ball was free, i.e., there was no limit on the number of simultaneous touches per player; and (b) defensive phase – individual marking was adopted, with pressure from the player exercising the specific principle of containment (defender) on the opposing player (ball carrier). Based on these restrictions and conditions, the tasks were structured as described in Figure 1.

The order of the tasks was randomly defined using the digital tool random.org (School of Computer and Statistics, Trinity College, Dublin, Ireland). The order of the tasks was as follows: T5 in week 2, T6 in week 3, T2 in week 4, T4 in week 5, T1 in week 6, and T3 in week 7. Each team experienced one experimental task per week during the training sessions. The data were collected between 6 pm and 9 pm, in the following order: U15 (Tuesdays), U17 (Mondays), U19 (Tuesdays) and Senior (Mondays). The training sessions had a total duration of 36 minutes, structured temporally as follows: (i) a standardized warm-up lasting 10 min, with the execution of tactical-technical actions with the ball: passing, receiving, ball control, dribbling and shooting, (ii) practice of the task over 16 minutes, divided between action and recovery; the total action time was 8 minutes, divided into 4 repetitions lasting 2 minutes, and the total recovery time was 8 minutes, with a 2-minute recovery interval between repetitions, and (iii) stretching exercises lasting 10 minutes. Before starting each training session, the state of readiness and recovery of participants was verified through the Total Quality Recovery Scale (TQR) using a 0- to 10-point scale [30]. Players with a TQR above 5 (“adequate recovery”) were considered fit to participate in the training tasks.

### Data collection

#### Sample characterization

To characterize the sample, the following measures were applied; (i) anthropometric data (height, measured through a stadiometer with a scale of 0.5 cm (Cescorf, Porto Alegre, Brazil); body mass, through a digital scale with 0.5 g (Omron HPF214, Kyoto, Japan); and skinfolds, through an adipometer with a scale of 0–60 mm and a resolution of 1 mm (Lange, Beta Technology, Santa Cruz, United States)); (ii) body composition (measured using thicknesses of three skinfolds: chest (CH), abdominal (AB), and thigh (TH) [5] and following the recommendations of the American College of Sports Medicine – ACSM [31]); (iii) body density (BD), using the predictive equation proposed and validated by Jackson and Pollock [32]; and (iv) body fat (% BF), estimated using the predictive equation proposed by Siri [33].

### Physical performance – external load

The effect of the tasks on the external load variables was measured with an inertial measurement unit (IMU; Oliver, Barcelona, Spain). The actions were recorded using IMU hardware, with a work frequency of 27 Hz, with 27 records per second (Recorder and Analyzer, Barcelona, Spain). The accuracy and reliability of these devices have been previously reported and validated [7]. From the positional data, variables were extracted based on two main external load categories: kinematic and mechanical [34]. The absolute and relative high-intensity actions were measured and recorded (Table 1).

**TABLE 1:** External Load variables measured.

Category	Variable	Units	Description
**Kinematics**	Total distance (TD)	Meters (m)	Total distance covered
	Relative distance (RD)		[12.1–18 Km/h] [> 18.1 Km/h]

**Mechanical**	Total distance at High Intensity Accelerations (HIAC)	Meters (m)	> 3 m/s^2^
	Total distance at High Intensity Decelerations (HIDC)		< -3 m/s^2^

### Statistical analysis

To analyse the external load, the field players were included (n = 48). Descriptive analysis was used to calculate the mean and standard deviation of the variables considered. The Shapiro-Wilk test was utilized to verify the normality of the data. One-way analysis of variance (ANOVA) was used to compare the characteristics between the categories with the Bonferroni post-hoc test. Mixed factorial ANOVA with repeated measures was adopted to compare tasks and teams (Task × Category) with the Bonferroni post-hoc test. The effect sizes were expressed as partial eta square (n^2^_p_). Significance was set at *p* ≤ 0.05. The effect size (ES) was calculated using Cohen’s d to compare the SSGs; it was classified as trivial when less than 0.10, small between 0.10 and 0.30, medium between 0.30 and 0.50, and large when greater than 0.50, as suggested by Fröhlich et al. [35]. All analyses were performed using IBM SPSS Statistics (IBM Corporation, Armonk, NY, USA, 25.0).

## RESULTS

The results for the total distance covered in metres showed significant interactions between the age categories and game formats (F (15.00) = 4.234; *p* < 0.001; n^2^_p_ = 0.261). For all the ages the high values were generally observed for T2 and the low values for T5 (see table 2 for detailed information about statistical differences between game formats). When compared the age categories, in relation to T6 (game format), higher values were observed for U15 (p = 0.020; ES = 1.10). In opposition, in the other game formats the opposite was observed, with the older players revealed higher values of total distance than the younger. In T4 format, U19 showed higher values than U15 (*p* = 0.005; ES = 0.50). In T5, higher values were observed in the Senior age category compared to U15 (p = 0.001; ES = 2.43) and U17 (p = 0.039; ES = 1.22), and U19 and U17 were higher than U15 (p = 0.001; ES = 2.08; p = 0.048; ES = 1.20), as shown in Table 2.

**TABLE 2:** Comparisons of the effect of manipulating the playing space and the number of players on kinematic variables according to age-category (n = 48).

	GK + 2 × 2 + GK		GK + 3 × 3 + GK		GK + 4 × 4 + GK	

	20 × 20	40 × 20	20 × 20	40 × 20	20 × 20	40 × 20

	T1	T2	T3	T4	T5	T6

	Mean±SD	Mean±SD	Mean±SD	Mean±SD	Mean±SD	Mean±SD
**Total distance (m)**						
U15	1042.7 ± 85.8^§*Ħ*^	**1215.6 ± 96.6** ^¶ § # *Ħ* ¥^	901.9 ± 105.5^*Ħ*^	1047.6 ± 95.4^§*Ħ*^	770.6 ± 95.9	1030.9 ± 117.1^§*Ħ*^
U17	1007.2 ± 104.3^*Ħ*^	**1202.8 ± 147.7** ^¶ § *Ħ* ¥^	1029.3 ± 158.9^*Ħ*^	1179.4 ± 125.6^¶§*Ħ*¥^	885.5 ± 96.6^*₸*^	1055.3 ± 137.7^*Ħ*^
U19	1026.2 ± 77.0	**1248.0 ± 97.6** ^¶ § *Ħ* ¥^	979.2 ± 42.1	1242.4 ± 51.0^¶§*Ħ*¥*₸*^	970.1 ± 90.4^*₸*^	1206.2 ± 123.9^¶§*Ħ₸*^
Seniors	1017.0 ± 107.3	**1202.0±65.1** ^¶ § *Ħ*^	1032.8 ± 97.2	1147.0 ± 174.4^¶*Ħ*^	1003.7 ± 82.1^*₸*ꞎ^	1091.3 ± 117.9

**12.1 – 18 Km/h (m)**						
U15	265.6 ± 91.7^§*Ħ*^	**362.8 ± 81.2** ^¶ § # *Ħ*^	157.2 ± 55.6	256.3 ± 51.7^§*Ħ*^	142.1 ± 56.1	330.0 ± 99.2^§*Ħ*^
U17	235.8 ± 46.8^*Ħ*^	**380.4 ± 85.3** ^¶ *Ħ*^	270.8 ± 102.9^*Ħ₸*^	349.2 ± 74.3^¶*Ħ*^	180.2 ± 61.0	347.9 ± 93.4^¶*Ħ*^
U19	260.0 ± 40.7	**407.8 ± 44.7** ^¶ § *Ħ*^	214.6 ± 32.2	342.7 ± 52.5^§*Ħ*^	232.1±64.7^*₸*^	352.1 ± 83.8^¶§*Ħ*^
Seniors	248.2 ± 74.9	**352.6 ± 47.0** ^¶ *Ħ*^	270.0 ± 56.9^*₸*^	324.8 ± 125.2^*Ħ*^	227.5 ± 83.7^*₸*^	324.4±88.6^*Ħ*^

**> 18 Km/h (m)**						
U15	17.5 ± 3.5	**90.1 ± 49.7** ^¶ #^	–	17.2 ± 5.7	–	73.3 ± 41.3^¶#^
U17	33.7 ± 18.4	**93.1 ± 40.0** ^¶ §^	45.7 ± 10.6	84.6 ± 25.9^¶§*₸*^	–	55.3 ± 24.6
U19	32.8 ± 18.7	**115.2 ± 28.9** ^¶ § # *Ħ*^	28.2 ± 10.8	66.5 ± 26.0^^§*Ħ₸*^^	32.5 ± 18.9	72.2 ± 25.8^§^
Seniors	44.0 ± 9.5	79.6 ± 20.3^§^	26.3 ± 16.4	**81.1 ± 37.5** ^¶ § *Ħ* *₸*^	20.1 ± 10.1	76.1 ± 47.6^¶§*Ħ*^

**Notes:** data are presented as mean and standard deviation. p-value obtained by mixed design factorial ANOVA. **Bold**: Higher result; Game formats:

¶ (p < 0.05) from T1;

*(p < 0.05) from T2;

§ (p < 0.05) from T3;

# (p < 0.05) from T4;

Ħ (p < 0.05) from T5; and

¥ (p < 0.05) from T6; Age-categories:

₸ (p < 0.05) from U15;

ꞎ (p < 0.05) from U17;

₶(p < 0.05) from U19;

ⱹ(p < 0.05) from Seniors.

The results for the relative distance covered at speeds ranging from 12.1 to 18.0 km/h revealed significant interactions between age categories and game formats (F (12.23) = 2.548; p = 0.004; n^2^_p_ = 0.175). For all the ages the high values were generally observed for T2 and low values for T3 (U17) and T5 (U15, U19 and senior) (see table 2 for detailed information about statistical differences between game formats). When compared the age categories, only differences were observed in T3 and T5 game formats. Again, the older players revealed higher values than the young players. In the T3 format, higher values were observed for U17 and Senior compared to U15 (p = 0.001; ES = 2.04; ES = 2.03) and, in T5, higher values were observed for U19 and Senior compared to U15 (p = 0.030; ES = 1.60; p = 0.044; ES = 1.52), as shown in Table 2.

The results for the relative distance covered at speeds above 18.0 km/h showed significant interactions between the age categories and game formats (F (15) = 2.691; p = 0.002; n^2^_p_ = 0.268). With the exception of senior players that revealed high values for T4, all other ages presented high values for T2. Absence of values (U15 and U17) and low values (U19 and senior) were observed for T5 (see table 2 for detailed information about statistical differences between game formats). When compared the age categories, only in the T4 format, higher values were observed in the U17, U19, and Senior categories compared to U15 (p = 0.001; ES = 11.82; ES = 8.65; ES = 11.21), as shown in Table 2.

The High Intensity Accelerations (HIAC) analysis revealed an interaction between age categories and game formats (F (15) = 2.095; p = 0.012 n^2^_p_ = 0.152). Interestingly, when the game formats were compared, only differences were observed for U15. T6 (the game format) present higher values of HIAC than T4 (p = 0.010; ES = - 1.45) and T5 (p = 0.013; ES = - 1.28). When compared the age categories, the older players tend to revealed higher values than the young players.In T3, higher values were observed for U17 and Senior age categories compared to U15 (p = 0.019; ES = 1.22; p = 0.008; ES = 1.34). In T4, U17, U19, and Senior age categories had higher values than U15 (p = 0.009; ES = 3.02; p = 0.004; ES = 3.30; p = 0.001; ES = 3.81) and, in T5, U19 had higher values than U15 (p = 0.024; ES = 2.23), as shown in Table 3.

**TABLE 3:** Comparisons of the effect of manipulating the playing space and the number of players on mechanical variables according to age-category (n = 48).

	GK + 2 × 2 + GK		GK + 3 × 3 + GK		GK + 4 × 4 + GK	

	20 × 20	40 × 20	20 × 20	40 × 20	20 × 20	40 × 20

	T1	T2	T3	T4	T5	T6

	Mean±SD	Mean±SD	Mean±SD	Mean±SD	Mean±SD	Mean±SD
**HIAC**						
U15	31.1 ± 15.4	38.0 ± 12.9^#Ħ^	26.5 ± 20.3	18.9 ± 9.6	22.6 ± 10.0	**49.0 ± 20.7** ^# Ħ^
U17	39.3 ± 13.8	42.8 ± 20.8	**51.3 ± 14.7** ^*₸*^	47.9 ± 15.8^*₸*^	36.4 ± 17.5	50.5 ± 22.7
U19	51.6 ± 22.5	**53.9 ± 13.9**	40.8 ± 9.4	50.6 ± 9.3^*₸*^	44.9 ± 12.2^*₸*^	45.4 ± 17.2
Seniors	44.5 ± 13.9	49.3 ± 19.1	53.7 ± 17.9^*₸*^	**55.5 ± 27.8** ^*₸*^	38.2 ± 20.6	44.0 ± 18.8

**HIDC**						
U15	52.3 ± 22.0	43.7 ± 18.6	49.5 ± 15.1	38.8 ± 13.6	37.7 ± 21.6	**64.1 ± 23.6** ^#^
U17	**73.5 ± 22.2** ^#^	57.6 ± 18.1	66.1 ± 16.4^*₶*^	50.8 ± 11.2	56.0 ± 22.1	68.4 ± 21.6
U19	**73.7 ± 22.1** ^§^	57.3 ± 6.6	44.9 ± 10.5	52.2 ± 8.5	52.6 ± 19.1	69.0 ± 20.9^§^
Seniors	59.4 ± 16.4	62.2 ± 19.0	**64.8 ± 17**.9^*₶*^	60.2 ± 19.7^*₸*^	59.0 ± 23.9	44.4 ± 13.8

**Notes:** data are presented as mean and standard deviation. p-value obtained by mixed design factorial ANOVA; Game formats:

¶ (p < 0.05) from T1;

* (p < 0.05) from T2;

§ (p < 0.05) from T3;

# (p < 0.05) from T4;

Ħ (p < 0.05) from T5); and

¥ (p < 0.05) from T6; Age-categories:

₸ (p < 0.05) from U15;

ꞎ (p < 0.05) from U17;

₶ (p < 0.05) from U19;

ⱹ (p < 0.05) from Senior

For High Intensity Decelerations (HIDC), there was an interaction between age categories and game formats (F (15) = 2.653; p = 0.002; n^2^_p_ = 0.181). Interestingly, only differences were observed in U15, with higher values for T6 (the game format) than for T4 (p = 0.006; ES = 1.86), in U17 with higher values for T1 compared to T4 (p = 0.041; ES = 2.03) and in in U19 with higher values for T1 (p = 0.002; ES = 2.74) and T6 (p = 0.013; ES = 2.30) than for T3. When compared the age categories, in the T3 format, the U17 and Senior categories had higher values than U19 (p = 0.021; ES = 2.02; p = 0.036; ES = 1.90) and, in the T4 format, the Senior category had higher values than U15 (p = 0.009; ES = 1.57), as shown in Table 3.

## DISCUSSION

The current investigation aimed to analyse the effect of manipulating the space and number of players in SSGs on the external load demands of futsal athletes of different age categories. The findings indicated that, independently of the age, structures with larger relative areas (133 m^2^ and 100 m^2^) generally present greater physical demands in kinematic variables. This aligns with the need for space to increase physical demands in the distance covered at different speeds. These results are consistent with previous studies in the literature that highlight an increase in external and internal load in tasks with large spaces and a low number of players, which stimulate speeds above 18 km/h [5, 36].

The same trend is observed when analysing the distance covered in larger relative and/or absolute areas, regardless of the category. However, we noted a tendency for U15 players to cover less distance in group structures of 3 and 4 players (from T3 to T6). These findings could also be supported by the previous studies, which suggest that smaller areas tend to increase the frequency of tactical-technical actions, thereby influencing the dynamics of the game [5, 19, 20].

Nevertheless, since this age category is associated with different levels of physical development and maturation [37], physical capacity differences among U15 players compared to other groups might be more pronounced. Variations in growth spurts, muscle development, and aerobic capacity can significantly influence performance metrics, such as distance covered and speed thresholds [38]. As a result, some U15 players may be at a disadvantage due to their ongoing physical development, which, along with the dynamic nature of the game and interpersonal coordination required [39], further contributes to the observed trends in mobility and game engagement characteristic of this age group.

Interestingly, concerning speeds above 18 km/h, we found that both player category and space constraints could be determining factors [22, 40]. Specifically, U15 players did not achieve speeds above 18 km/h in the 20 × 20 structures (T3 and T5), while U17 players only failed to reach these speeds in the T5 structure. Both findings could be explained by space constraints and game dynamics, considering the expertise level and the number of participants [5, 41].

Analysing mechanical variables, we observed greater variability in response to different age categories and tasks. For instance, in T1 (20 × 20), fewer players and reduced space may lead to more frequent interactions and quicker decision-making processes. This heightened action frequency demands rapid accelerations, decelerations, and changes in direction, placing significant strain on the player’s agility and explosiveness. On the other hand, the T6 (40 × 20) game format presents a different set of challenges. The increased space facilitates higher-intensity running speeds, potentially intensifying acceleration and deceleration demands. These results may also be related to the type of actions that occur according to the number of players. In this sense, a smaller number of players favours dribbling and 1 vs. 1 shooting actions, while a larger number of players in the task favours passing and ball retention actions, with collective tactical behaviour [10]. These results suggest that regardless of field size and number of players, relative area might not play as crucial a role in the analysis of the mechanical dimension. However, medium or large areas also promote high-intensity actions and can encourage tactical actions representative of the game [5, 42].

Interestingly, Ribeiro et al. [43] recently showed that acceleration actions are more related to the performance of actions without the ball (support movements and defensive trajectories), while decelerations are more related to the performance of ball actions (dribbling, passing, shooting). Linking the playing areas with the number of players and the more frequent actions that occur in each task, the results obtained are in line with previous research [16, 17]. Larger relative and absolute areas tend to promote more actions without the ball and particularly high displacements of players, while smaller playing areas seem to entail an increase in the frequency of accelerations and decelerations regarding the frequency of small displacements and also the frequency of actions with the ball. Overall, coaches can optimize small-sided games by using the constraints of space and the number of players to enhance player development and performance. For instance, sessions conducted in smaller spaces can prioritize agility drills, quick decision-making exercises, and ball control techniques. Conversely, training sessions in larger areas may emphasize spatial awareness and high-intensity displacements on the field.

However, the results of this study should be interpreted with caution, as despite analysing different age groups, an important limitation is that the study was conducted with only one team. Future research should consider evaluating different competitive levels and investigating the maturation of young athletes, to clarify some results according to expertise level, game understanding, and the maturation level of young futsal players.

## CONCLUSIONS

The design of the training is essential to prepare futsal players for the requirements of the competition, so the tasks should be analysed and selected appropriately according to the training objectives and days of the microcycle. Futsal coaches should consider specific references and adjust to the context of the age category, which allows better programming and control of the training load in an appropriate and individualized way. This research provides valuable insights for practitioners, highlighting several key practical applications: a) adjust the space and number of players to increase physical demands, particularly in larger playing areas (133 m^2^ and 100 m^2^); b) use medium or large areas to promote high-intensity actions, such as sprinting and rapid transitions; and c) utilize data from external load devices to individualize training loads according to player development and competition demands. The different age categories show a tendency to increase the magnitude of the kinematic variables as the age category of the futsal players increases, although exceptions are observed, mainly in the senior age category.
